# The Formulation of Bacteriophage in a Semi Solid Preparation for Control of *Propionibacterium acnes* Growth

**DOI:** 10.1371/journal.pone.0151184

**Published:** 2016-03-10

**Authors:** Teagan L. Brown, Steve Petrovski, Zoe A. Dyson, Robert Seviour, Joseph Tucci

**Affiliations:** 1 La Trobe Institute for Molecular Sciences, La Trobe University, Bundoora, VIC, Australia; 2 Department of Physiology, Anatomy and Microbiology, La Trobe University, Bundoora, VIC, Australia; University of Ulster, UNITED KINGDOM

## Abstract

**Aims:**

To isolate and characterise phage which could lyse *P*. *acnes* and to formulate the phage into a delivery form for potential application in topical treatment of acne infection.

**Methods and Results:**

Using standard phage isolation techniques, ten phage capable of lysing *P*. *acnes* were isolated from human skin microflora. Their genomes showed high homology to previously reported *P*. *acnes* phage. These phage were formulated into cetomacrogol cream aqueous at a concentration of 2.5x10^8^ PFU per gram, and shown to lyse underlying *P*. *acnes* cells grown as lawn cultures. These phage formulations remained active for at least 90 days when stored at four degrees Celsius in a light protected container.

**Conclusions:**

*P*. *acnes* phage formulated into cetomacrogol cream aqueous will lyse surrounding and underlying *P*. *acnes* bacteria, and are effective for at least 90 days if stored appropriately.

**Significance and Impact of the Study:**

There are few reports of phage formulation into semi solid preparations for application as phage therapy. The formulation method described here could potentially be applied topically to treat human acne infections. The potential exists for this model to be extended to other phage applied to treat other bacterial skin infections.

## Introduction

Acne is a widespread, chronic disease of the pilosebaceous unit, resulting in the formation of lesions on the face, neck and upper torso [1]. The aetiology of acne is multifactorial, but four pivotal processes contribute to its pathogenesis. These are an increased sebum production, altered maturation and migration of keratinocytes, an inflammatory response and follicular inhabitation by the native skin bacterium *Propionibacterium acnes* [2,3]. The excessive production of sebum is a critical factor, and this, as well as the over growth and differentiation of the keratinocytes, create an environment that allows *P*. *acnes* to increase in numbers and trigger inflammation [4].

*P*. *acnes* is a Gram positive, non-motile facultative anaerobic organism, which in excess numbers is believed to elicit an inflammatory response contributing to formation of acne lesions [5–8]. Type IA *P*. *acnes* strains have been isolated frequently from human skin samples [9–13], and studies suggest that clade IA_1_ isolates are the most prominent type seen in patients with acne [9–13]. Common first-line treatments for mild and moderate acne are topically applied antimicrobial chemicals, oral antibiotics or retinoids [14]. These topical agents are usually tolerated well, although some patients report dermal irritation, scaling or itching following their application. In moderate acne infections, oral antibiotics are taken for periods of up to six months, with the distinct possibility of specific side effects, and interactions with other prescribed medicinal drugs [15]. Furthermore, there is global concern that antibiotic resistant strains of *P*. *acnes* strains are becoming increasingly common [16–21], a problem which is exacerbated by prolonged and extensive antibiotic use. For more severe and unresponsive cases, oral retinoids may be prescribed. However, an important contra-indication when using these drugs is that they are teratogenic, and apart from this, they may also produce other adverse reactions, which include dyslipidaemia, altered blood glucose levels, eye and skin disorders and mood disorders [15].

An alternative to antibiotics is to use lytic bacteriophage (phage), entities which are endogenous killers of bacterial cells [22,23]. Their discovery dates back to the beginning of the 20^th^ century, but until recently their therapeutic application and exploitation, although common in former Eastern bloc countries, was limited because of widespread and at that time successful use of antibiotics in Western medicine to treat bacterial infections [24–26]. There are several reasons why using phage to treat bacterial infections is attractive. They are highly specific, lysing only their bacterial hosts, and are auto “dosing” in that phage replication at the site of infection leads to marked increases in their titre. Furthermore, phage are generally regarded as medically and environmentally safe, bring about minimal disruptions to the autogenous microbial community, and will lyse antibiotic resistant strains [27]. In 2014, the National Institutes of Health in the U.S. suggested that the use of phage was among one of the more innovative and promising components of strategies to combat antimicrobial resistance [28].

Successful application of phage therapy in humans has been reported with a phage impregnated polymer, shown to be effective in treating *Pseudomonas*, *Staphylococcus and Escherichia* infected ulcers and poorly healing wounds [29–31]. Chronic otitis attributed to *Pseudomonas aeruginosa* has been treated with phage in clinical trials, with favourable therapeutic outcomes [32]. Topical application of phage was successful in treating *P*. *aeruginosa* infections in animal models [33–35], and in humans, phage have been prepared in an “antiseptic” gel and a paraffin oil-based lotion, specifically to target *Acinetobacter baumannii* infections [36]. Formulations of lyophilised phage have been used as a nasal spray for treatment of bacteria in the nasal passage [37], and as inhalable powders for treatment of pulmonary infections [37–39].

To date, however, there have been scant adequately controlled reports of the formulation of phage into dosage forms that allow for topical application for the treatment of human skin infections such as acne vulgaris.

The isolation and genome sequencing of a number of phage for *P*. *acnes* has been reported [40–43]. Liu et al (2015) have recently characterised a further 48 *P*. *acnes* phage, and suggested that the phage can adopt a pseudolysogenic lifecycle in clade 1A_1_ *P*. *acnes* strains. In this study we have isolated, sequenced and characterised ten additional *P*. *acnes* phage. These were then formulated into a semi solid aqueous cream base, and applied to type IA_1_ *P*. *acnes* lawn cultures *in vitro*. Under the conditions used here, they lysed the underlying *P*. *acnes* cells. These phage cream formulations were most stable if stored at 4°C in light protected containers, where their lytic properties were maintained for at least 90 days.

## Materials and Methods

### Bacterial strains and culture conditions

All cultures were grown using Reinforced Clostridial Medium (RCM) containing 1% peptone (Oxoid, Adelaide, Australia), 1% ‘lab lemco’ powder (Oxoid), 0.5% glucose (Sigma, Sydney), 0.5% sodium chloride (Sigma), 0.3% yeast extract (Oxoid), 0.3% anhydrous sodium acetate (Chem-supply, Gillman, Australia) and 0.05% soluble starch (Difco, Detroit, USA). Agar 1.5% (w/v) was added when solid media were required and all cultures were grown anaerobically using the Anaerogen system (Oxoid) at 37°C.

Ten *P*. *acnes* isolates were obtained from facial skin swabs of humans and transferred onto RCM agar. The procedure for sampling involved self-swabbing (with sterile swabs) by participants wearing protective gloves and laboratory coats. Swabs were then immediately placed into sterile tubes and collected. All samples were obtained with oral consent, (this was documented in both hard copy and soft copy files, stored in the Department of Pharmacy and Applied Sciences, La Trobe University) de-identified and handled according to the La Trobe University Ethics committee. No ethical concerns were raised as the human material was not the focus of the study and bacteria obtained were not traced back to the individual. For unequivocal identification, PCR amplification of their 16S rRNA genes was performed using universal primers [44]. PCR products were then processed using an Ultra Clean^®^ DNA purification kit (MO-BIO, California USA), and the DNA analysed by Sanger sequencing at the Australian Genome Research Facility (Brisbane, Australia). *P*. *acnes* strains were biotyped according to the Single-Locus Sequence Type (SLST) scheme as described previously [13]. The *P*. *acnes* type strain (ATCC 6919), together with additional *Propionibacterium* species: *P*. *acidipropionici* (ATCC 25562), *P*. *avidum* (ATCC 25577), *P*. *cyclohexanicum* (ATCC 700429), *P*. *jensenii* (ATCC 4964) and *P*. *thoenii* (ATCC 4872), all obtained from the American Tissue Culture Collection (Washington DC, USA), were used in this study, along with *P*. *freudenreichii* (a gift from CHR Hansen, Denmark).

### Isolation and purification of phage

Individual broth cultures of the ten *P*. *acnes* strains isolated from facial skin swabs (described above) were incubated for two days. These cultures were centrifuged (800 ***g*** for 5 min) to remove bacterial cells and the supernatants filtered through 0.2 μM cellulose acetate filters (Advantec, Melbourne, Australia). 10 μL of the filtrate was then placed onto a lawn of the *P*. *acnes* strain that was initially used to inoculate the broth. The presence of phage was identified by plaque formation on the bacterial lawn. Individual plaques were excised along with the underlying agar, placed into 600 μL of broth and vortexed to allow solubilisation of the phage into the broth. After centrifugation (15,000 ***g*** for 2 min) the supernatant was serially diluted (1:10) so that decreasing concentrations were obtained, to a concentration of 10^−8^. 10 μL of each dilution was spotted onto a lawn of *P*. *acnes* to obtain single plaques. The purification process was repeated four times to ensure that each plaque was the result of one virion infection. In these experiments, each phage was always propagated on the host it was obtained from. A phage stock was generated by suspending an individual plaque (as described in the purification process above) in 50 mL of broth inoculated with 1 mL of an exponential culture of *P*. *acnes* using a Multiplicity of Infection of 0.1 PFU per bacterium, for 24 hours. Following incubation, the culture was centrifuged (800 ***g*** for 5 min) and the supernatant filtered through a 0.2 μM cellulose acetate filter (Advantec). To assess the concentration of this phage stock (PFU mL^-1^), serial dilutions were performed as described above.

### Phage host range

The phage stock at concentrations of approximately 10^10^ PFU mL^-1^ were serially diluted 10-fold and a 10 μL aliquot of each dilution was spotted directly onto a lawn culture of each of the *Propionibacterium* strains listed above. The observation of plaques on the lawn culture indicated that the phage was able to infect and lyse the host.

### Determination and characterisation of pseudolysogeny in clade 1A_1_ *P*. *acnes* strains

In order to investigate whether PAC1-PAC10 phage genomes exist in a circular form, and given that our PAC phage are identical across the sequence recognised by the PCR primers (see below), we performed the same PCR experiment as Liu et al. (2015) when they assessed the topology of their *P*. *acnes* phage genomes. The primers used were: {Forward} 5’-CGAAGCCGACCACATCACACC-3’, {Reverse} 5’-TCATCCAACACCTGCTGCTGCC-3’ and PCR conditions were 94°C for 5 min, then 35 cycles of 94°C for 45 s, 53°C for 35 s, 72°C for 1 min, then 72°C for 10 min. PCR products were then processed using an Ultra Clean^®^ DNA purification kit (MO-BIO, California USA), and the DNA analysed by Sanger sequencing at the Australian Genome Research Facility (Brisbane, Australia).

To test for immunity to subsequent phage lysis, resistant host cells taken from plaque centres developed following PAC1 to PAC10 phage lysis of *P*. *acnes* ATCC 6919 were subcultured twice. Bacterial lawns of the resistant strains were then spotted with 10 μL aliquots of all the PAC phage (at a concentration of 1x10^8^ PFU mL^-1^), and subsequent cell lysis recorded. For assessing whether phage genomes were organised circularly, possibly as episomes within these resistant *P*. *acnes* strains, the same PCR reaction as described above were performed on DNA isolated from these bacteria [44].

### Isolation of phage DNA

Five mL of phage stock (>10^9^ PFU mL^-1^) was treated with MgCl_2_ (final concentration 5 mmol L^-1^), DNase I and RNase A (final concentrations of 10 μg mL^-1^) for 30 min at room temperature. Virions were recovered by polyethylene glycol (PEG) precipitation using PEG 8000 and sodium chloride (final concentrations 10% [w/v], and 1 mol L^-1^ respectively) at 4°C overnight [45]. The precipitate was centrifuged (17,000 ***g*** for 15 min) and the pellet resuspended in nuclease free water (Promega, Sydney, Australia). Phage were exposed to Proteinase K (final concentration 50 μg mL^-1^), EDTA (final concentration 20 mmol L^-1^) and sodium dodecyl sulphate (final concentration of 0.5% [v/v]) at 55°C for 1 h. After incubation an equal volume of phenol-chloroform-isoamyl alcohol (29:28:1) was added and thoroughly mixed before being centrifuged (12,000 ***g*** for 3 min). The aqueous phase was carefully removed and an equal volume of isopropanol, and 1/10 volume 3 mol L^-1^ sodium acetate (pH 5) was added. The mixture was left at -20°C overnight to allow DNA precipitation. After centrifugation (12,000 ***g*** for 10 min) the supernatant was removed, the DNA pellet was washed with 70% ethanol, air dried and resuspended in 25 μL of nuclease free water (Promega, Sydney, Australia).

### Phage genome sequencing and annotation

Extracted phage DNA was prepared for sequencing using a Nextera^®^ XT DNA sample preparation kit as per the manufacturer’s instructions. The DNA libraries were sequenced using a Miseq^®^ V2 reagent kit (300 cycles) on an Illumina MiSeq^®^ as 150 bp paired end reads. Sequenced reads were assembled *de novo* using CLC workbench, version 7.5.2 (CLC Bio-Qiagen, Denmark), and open reading frames predicted using Glimmer, version 3.02 [46]. ARAGORN [47] and tRNAscan-SE [48] were used to predict tRNA and tmRNA present in the sequences. Alignments of phage genomes were performed using Mauve [49].

### Phage phylogenetics

The genomic sequences of the phage isolated here were compared with sequences of the 62 previously reported *P*. *acnes* phage genomes (S1 Table). Their phylogenetic relationships were determined by the Neighbour Joining method, utilising the Jukes-Cantor algorithm, within the CLC Genomics Workbench (version 7.5.2). Bootstrapping was performed on 1000 replicates.

### Formulation of phage cream

For each of the ten phage isolated here, a phage suspension of 5.0x10^9^ PFU mL^-1^ in phosphate buffered saline (PBS) (137 mmol L^-1^ NaCl, 2.7 mmol L^-1^ KCl, 10 mmol L^-1^ Na_2_HPO_4_ and 1 mmol L^-1^ KH_2_PO_4_), was used for formulation into cetomacrogol cream aqueous (Biotech Pharmaceuticals, Melbourne, Australia), a non-ionic cream. This choice followed testing a range of non-ionic cream bases, since it was considered that these were least likely potentially to interact with the phage particles. Therefore, each phage cream produced contained a single phage isolate (PAC1 to PAC10) formulated into this cream. Phage were mixed by gradual serial addition to the cream [50] to a final concentration of 2.5x10^8^ PFU per gram. The method of serial addition is a standard pharmaceutical formulation technique: in a sterile hood, using a sterile stainless steel blade spatula on a sterile glass slab, the process describes the addition, mixing and even distribution of phage solution into a small portion (approximately 2 g) of the cream, then thorough mixing of this small portion with an equal mass of fresh cream. This process is repeated until all the fresh cream has been incorporated, and the medicament (in this case, the phage) is evenly dispersed throughout the cream.

To test whether they were capable of lysing their host bacteria in this formulation, an RCM agar lawn plate of each *P*. *acnes* strain was prepared and 0.5 cm^3^ of each of the various PAC1 to PAC10 phage creams were syringed onto the surface. The plates were incubated at 37°C for 2 days and any lysis of the bacteria in the presence of the cream was indicated by a clear zone.

The lowest concentration of phage required to elicit lysis was also determined, using a ten-fold dilution series of the phage suspension in PBS. The dilution series was then formulated into cream as above, such that the formulated phage concentrations were between 5.0 x 10^8^ PFU per gram and 5.0 PFU per gram of cream.

### Stability of lytic phage formulation

To ascertain whether the phage remained active in the cream, a portion of cream formulated with PAC1, at 2.5x10^8^ PFU per gram, was tested for its thermo-stability and photo degradation. The cream was stored at temperatures of 4°C, 20–25°C and 45°C in light protected bottles, and exposed to 50 Lux of light, the standard illumination of a typical room, at 20–25°C. The capacity of the phage to lyse the underlying bacteria following these treatments was assessed by methods described above at weekly intervals up to 90 days. For a quantitative assessment of the phage lytic activity and stability following storage of the creams at various temperatures and light exposures, a 0.05 g sample of the phage cream was taken at weekly intervals for up to 60 days. This sample was mixed into PBS to a volume of 1 mL, vortexed well and then centrifuged (12,000 ***g*** for 10 min). A dilution series of an aliquot of the supernatant was then performed, and 10 μL of these various dilutions then placed onto a lawn of the cutaneous *P*. *acnes* strain that PAC1 was isolated from, to ascertain the numbers of active phage (given as PFU per gram of cream).

### Nucleotide sequence accession numbers

Genomic nucleotide sequences of the phage described here (PAC1 to PAC10) were deposited into GenBank under accession numbers KR902978, KR902979, KR902980, KR902981, KR902982, KR902983, KR902984, KR902985, KR902986 and KR902987.

## Results

### Isolation, characterisation and general features of *P*. *acnes* bacteriophage

**Fig 1:**
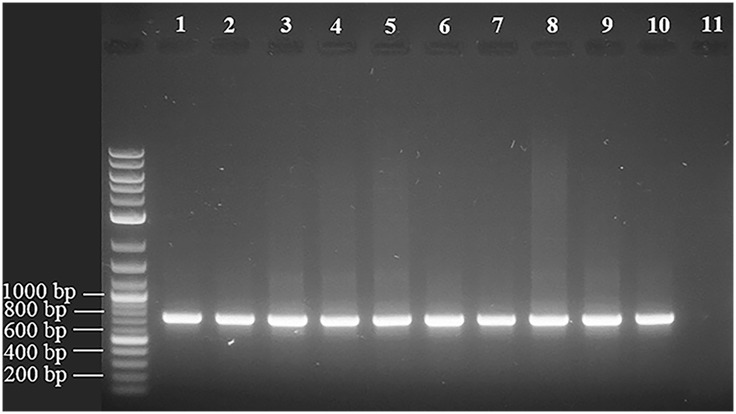
PAC1 to PAC10 phage are able to circularise. PCR was performed across a region incorporating the 11 bp overhang at the 3’ end of the genome. A band of approximately 735 bp was seen in all the phage and was only possible if the genomes were circular. Lanes 1 to 10 represent DNA from PAC phages and lane 11 is *Propionibacterium freudenreichii* prophage DNA as negative control.

Ten cutaneous *P*. *acnes* strains were isolated from human skin and biotyped with SLST as either group A1, A2 or E8, all of which correspond to the clade IA_1_ [13]. Type IA *P*. *acnes* strains have been isolated frequently from human skin samples, and type IA_1_ isolates are found commonly in patients with acne [9–13]. Ten phage were isolated from these strains and are referred to here as PAC1 to PAC10. While the genomes of the phage described here display up to 96% similarity at the nucleotide level with other reported *P*. *acnes* phage [40–43], there are some notable differences as described below. The genome sequence data for each phage were assembled *de novo* with an average coverage greater than 2000 times, as detailed in Table 1. The ten dsDNA phage genomes ranged in size from 29,428 bp to 29,786 bp. Each genome contained an eleven base pair overhang at the 3’ end of the genome (5’–TCGTACGGCTT), as described for other characterised *P*. *acnes* phage [42]. As reported previously, the overhangs may facilitate the circularisation of their genomes. To determine whether this was the case with all ten PAC phage, and given that their sequences in these regions were homologous to other *P*. *acnes* phage, we performed PCR across these regions using the same primers and conditions described by Liu et al (2015). We observed a similar band of approximately 735 bp in each of the ten PAC phage, suggesting circularisation was likely (Fig 1). These PCR products were sequenced and confirmed to be DNA corresponding to the expected regions in the phage genomes.

**Table 1:** PAC1 to PAC10 genomic features and nucleotide similarity to P101A phage.

Phage	Genome length	Average Coverage	% GC content	Predicted *orf*s	Similarity to P101A
PAC1	29,605 bp	6,560 x	54.0	43	96%
PAC2	29,602 bp	3,578 x	54.0	44	96%
PAC3	29,526 bp	4,332 x	54.0	43	96%
PAC4	29,581 bp	3,654 x	54.0	44	95%
PAC5	29,428 bp	3,269 x	54.2	44	95%
PAC6	29,609 bp	8,152 x	54.1	43	94%
PAC7	29,551 bp	3,999 x	54.1	43	94%
PAC8	29,488 bp	2,324 x	54.0	43	94%
PAC9	29,786 bp	2,843 x	54.0	44	95%
PAC10	29,704 bp	6,489 x	54.0	44	94%

**Fig 2:**
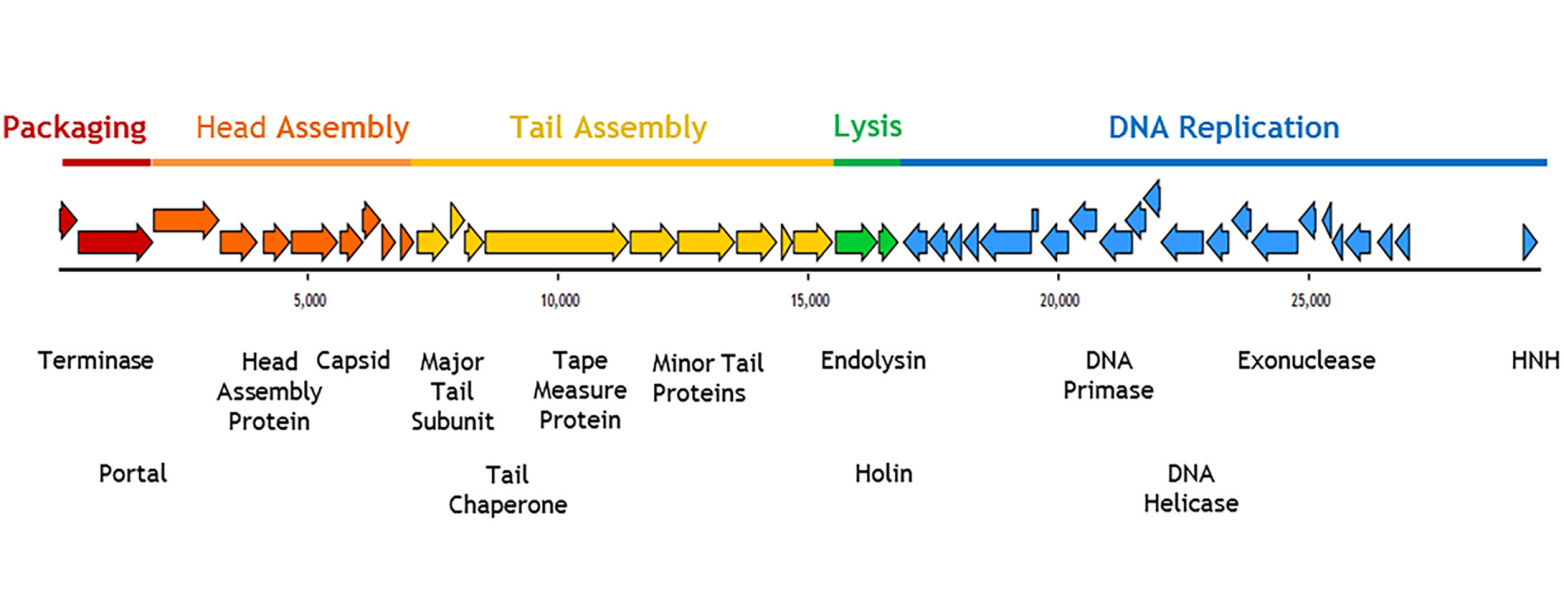
Modular structure of PAC1. The coloured arrows indicate predicted ORFs and direction of translation. Modular structure is indicated in blocks of colour and the putative gene function is noted below.

**Fig 3:**
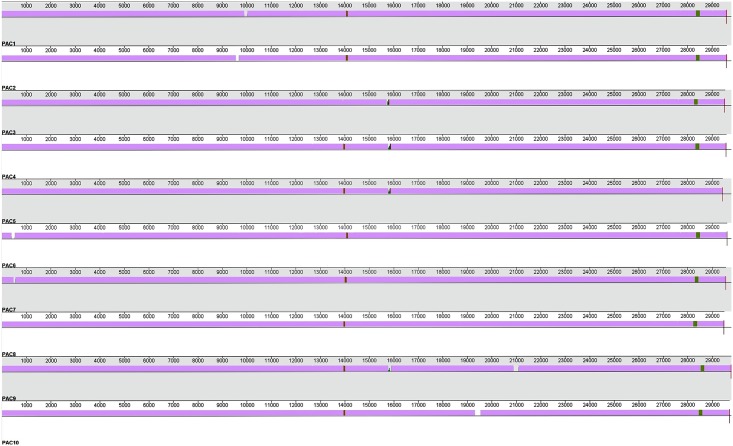
Alignment of genomes PAC1 to PAC10 using Mauve alignment program. Mauve colour indicates regions of conserved sequence between all phage, ‘gaps’ indicate insertions in a given phage genome and the regions of colour indicate sequence similarity across some phage and not others.

The G+C mol% content of the phage genomes covers a narrow range of 54.0 to 54.2, which is lower than that of their host *P*. *acnes* genome (~60%; see Table 1). When these genomes were annotated each had a modular structure with 43 or 44 predicted open reading frames (*orf*s) (see Fig 2; Table 1). No putative tRNA or tmRNA regions were predicted. The predicted proteins from these *orf*s were similar to those reported for previously published *P*. *acnes* phage [40–43]. Importantly, none of the phage genomes described here contained any bacterial virulence factors. Annotation and alignment of these ten genomes revealed that they shared greater than 98% similarity (Fig 3). Regions of non-homologous recombination were present among them, a common feature of other *P*. *acnes* phage [42]. Table 1 shows that the genomes of the ten PAC phage range from between 94% to 96% homology to the most similar previously sequenced *P*. *acnes* phage P101A [42]. Fig 3 shows the sequence similarity of the ten phage genomes to each other.

### PAC phage genomes

The ten PAC phage displayed slight modifications to the individual *orf*s encoding their major structural and replicative functional proteins. Genomic differences among the 10 PAC phage include the following. PAC1 phage genome had a 117 bp insertion, located at 9,956 bp (Fig 3). This insert occurred in the middle of the putative tape measure protein-encoding *orf*, presumably elongating its polypeptide product. Any modification to its function is unknown. The PAC2 phage genome had a 115 bp insertion located at 9,595 bp (Fig 3), again within the putative tape measure protein encoding *orf*. This insert creates effectively two smaller truncated *orf*s. One encodes a polypeptide 585 amino acids in length, and almost 100% homologous to a segment of the tape measure protein-encoding *orf* in *P*. *acnes* phage PHL132N00 [43]. The other *orf*, encoding a protein of 375 amino acids, is almost 100% homologous to the tape measure protein *orf* in *P*. *acnes* phage PHL060L00 [43]. Such data suggest a clear evolutionary linkage between the tape measure proteins in these three phage, probably following a recombination event between PHL132N00 and PHL060L00 phage.

The PAC3 phage had a 69 bp deletion contained in the *orf* encoding the putative minor tail protein. Consequently, part of the region encoding the H-type lectin domain (pfam09458) which may represent a tail protein allowing initiation of binding to bacterial cell walls [41], is missing. This phage genome also has a 105 bp insert located at 15,726 bp (Fig 3) in the putative endolysin encoding *orf*, thus elongating the polypeptide product by 35 amino acids. While the functional effect of possessing this insert is not known, it does not alter the encoding of the amidase domain (pfam01510), whose role is fundamental in cleaving amide bonds in bacterial cell wall peptidoglycans [51]. The PAC4 phage genome also has a 94 bp insert located at 15,793 bp (Fig 3) in the putative endolysin encoding *orf*. This creates a stop codon, and divides this sequence into two *orf*s. While the product of one *orf* retains the region encoding an intact amidase domain, and is 98% homologous to a putative endolysin from *P*. *acnes* phage PHL150M00 [43], it is unclear what the effect of truncation of this protein is. A similar insert occurs at the same site in both the PAC5 and PAC9 phage genomes, generating the same two distinct *orf*s. Thus this region may be a “hot spot” representing a location where insertion events are more likely to occur. Liu et al (2015) have suggested that the endolysin gene may represent a location in the genome that undergoes rapid evolution. Such alterations to the phage endolysin gene may affect bacterial cell wall hydrolysis. The PAC9 phage genome also has a 185 bp insertion located at 21,075 bp (Fig 3) in its putative DNA primase *orf*. Consequently this modified DNA primase-encoding *orf* shares only 42% homology to that of *P*. *acnes* phage PHL092M00 DNA primase-encoding *orf* [43], its closest matching sequence. While the region for the Toprim encoded domain (pfam13662) of the DNA primase gene, which is crucial for the initiation steps in DNA replication [52], is intact within this truncated *orf*, any functional outcomes of this alteration are unknown.

### Phylogenetic comparisons between PAC phage and other *P*. *acnes* phage

**Fig 4:**
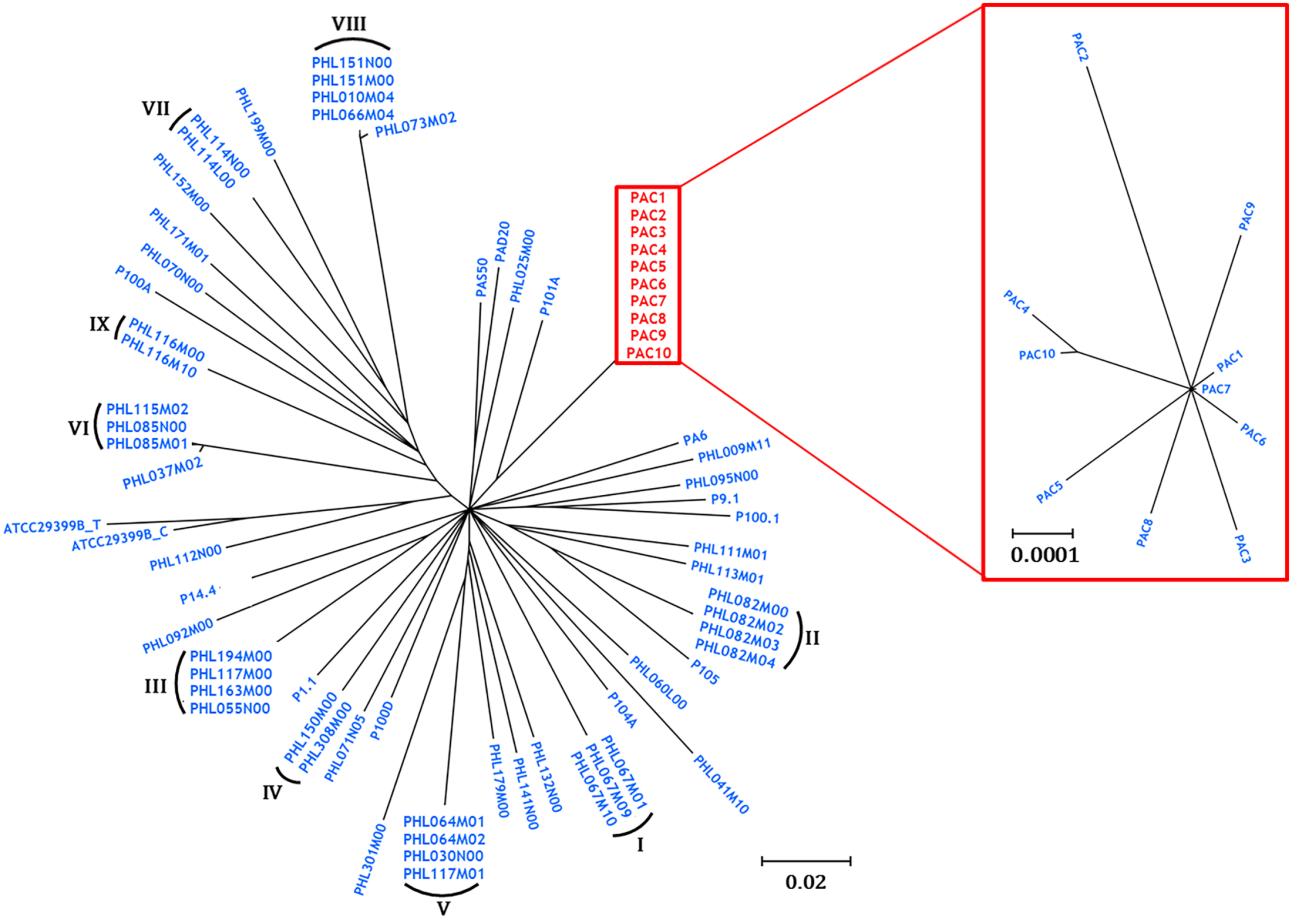
Phylogenetic tree of the 72 known sequenced P. acnes phages. When bootstrap values <80 were collapsed, 53 distinct strains emerged, with the PAC phage effectively adding another ten strains to the 43 reported previously. Of these, nine highly homologous groups, with members differing by as little as 11–14 base pairs, emerged as distinct clonal strains (strains I-IX), as reported recently (33). Insert shows phylogenetic relationships between PAC1 to PAC10.

The genomes of the PAC phage were compared to all 62 previously sequenced *P*. *acnes* phage [40–43], and their phylogenetic relationships established. The ten PAC phage reported here are most closely linked evolutionarily to the phage P101A (Fig 4).

### Lytic capacity of the *P*. *acnes* phage

**Fig 5:**
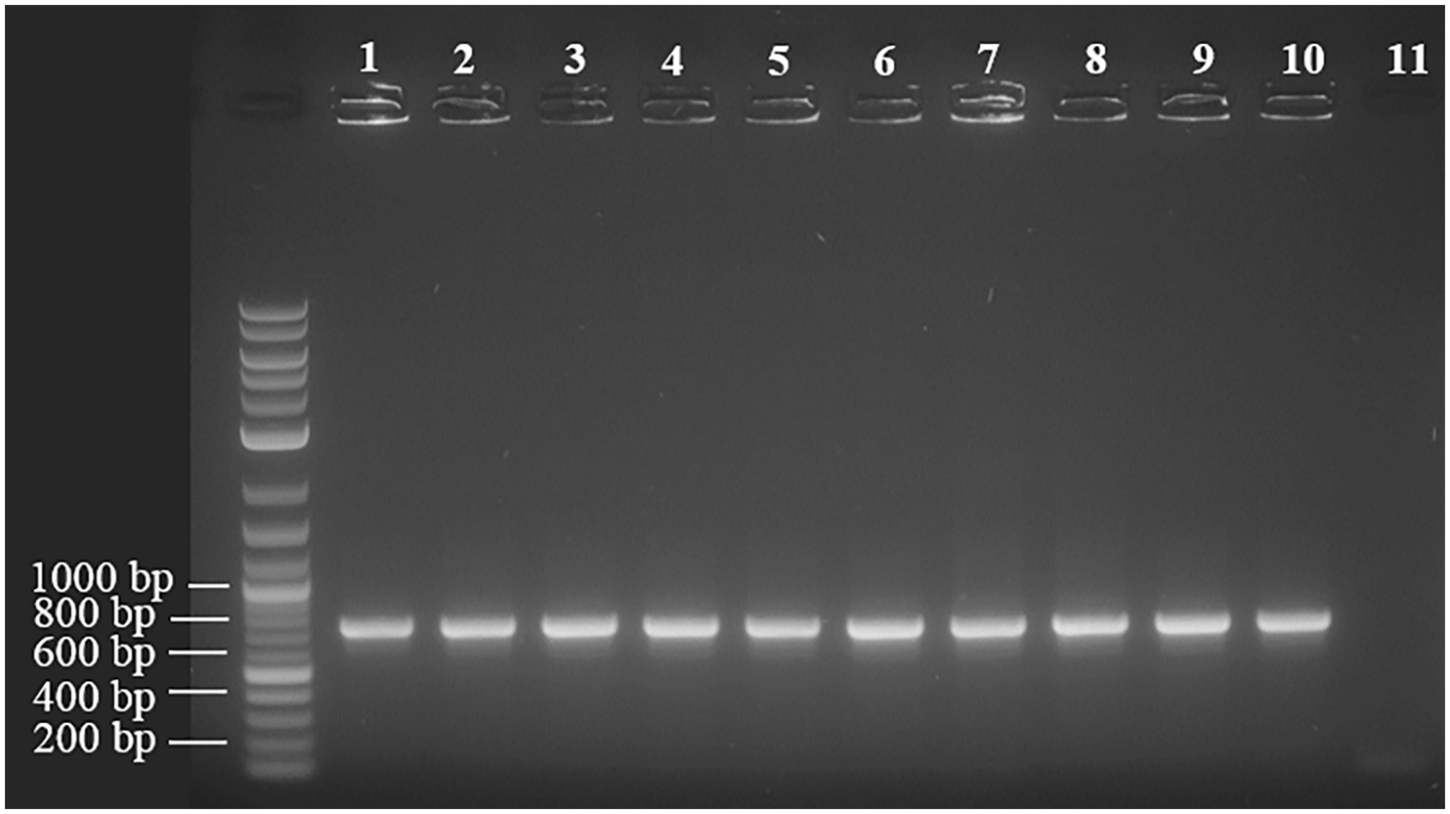
DNA was extracted from the phage resistant subcultured cells derived from the regrowth area within plaques on *P*. *acnes* (ATCC 6919). PCR across the 3’ phage genome overhang displayed a band of approximately 735 bp, potentially indicating the presence of circularised phage episomal DNA. Lanes 1–10 represent DNA extracted from resistant *P*. *acnes* (ATCC 6919) strains arising from exposure to PAC1-PAC10. Lane 11 represents control DNA extracted from *Propionibacterium freudenreichii*.

The lytic capacity of phage PAC1 to PAC10 was limited to the ten cutaneous *P*. *acnes* strains isolated here and *P*. *acnes* (ATCC 6919), with each phage lytic against all of the cutaneous and the *P*. *acnes* (ATCC 6919) strains. They did not lyse *P*. *acidipropionici* (ATCC 25562), *P*. *avidum* (ATCC 25577), *P*. *cyclohexanicum* (ATCC 700429), *P*. *jensenii* (ATCC 4964), *P*. *thoenii* (ATCC 4872) or *P*. *freudenreichii*. However, as also reported by Liu et al. (2015), *P*. *acnes* (ATCC 6919), which belongs to the IA_1_ clade, showed a regrowth of cells originating from the centre of the plaque, following its initial formation. This event was seen when each of ten PAC phage were used to infect this strain. It did not occur, however, when the other cutaneous *P*. *acnes* type IA_1_ strains we isolated were infected with the ten PAC phage. Clear plaques (from which no regrowth was seen, nor from which any bacteria could be subcultured) resulted from their lytic cycles with these strains. As with the events described by Liu et al. (2015), subcultured cells from the regrowth areas within the *P*. *acnes* (ATCC 6919) plaques showed resistance to other *P*. *acnes* phage upon subsequent exposure. The presence of circularised phage DNA from these regrown cultures (Fig 5) suggested they may exist as intracellular episomes [43]. These PCR products were sequenced and confirmed to be DNA corresponding to the expected regions in the phage genomes.

### Lytic capacity and stability of the phage cream formulations

**Fig 6:**
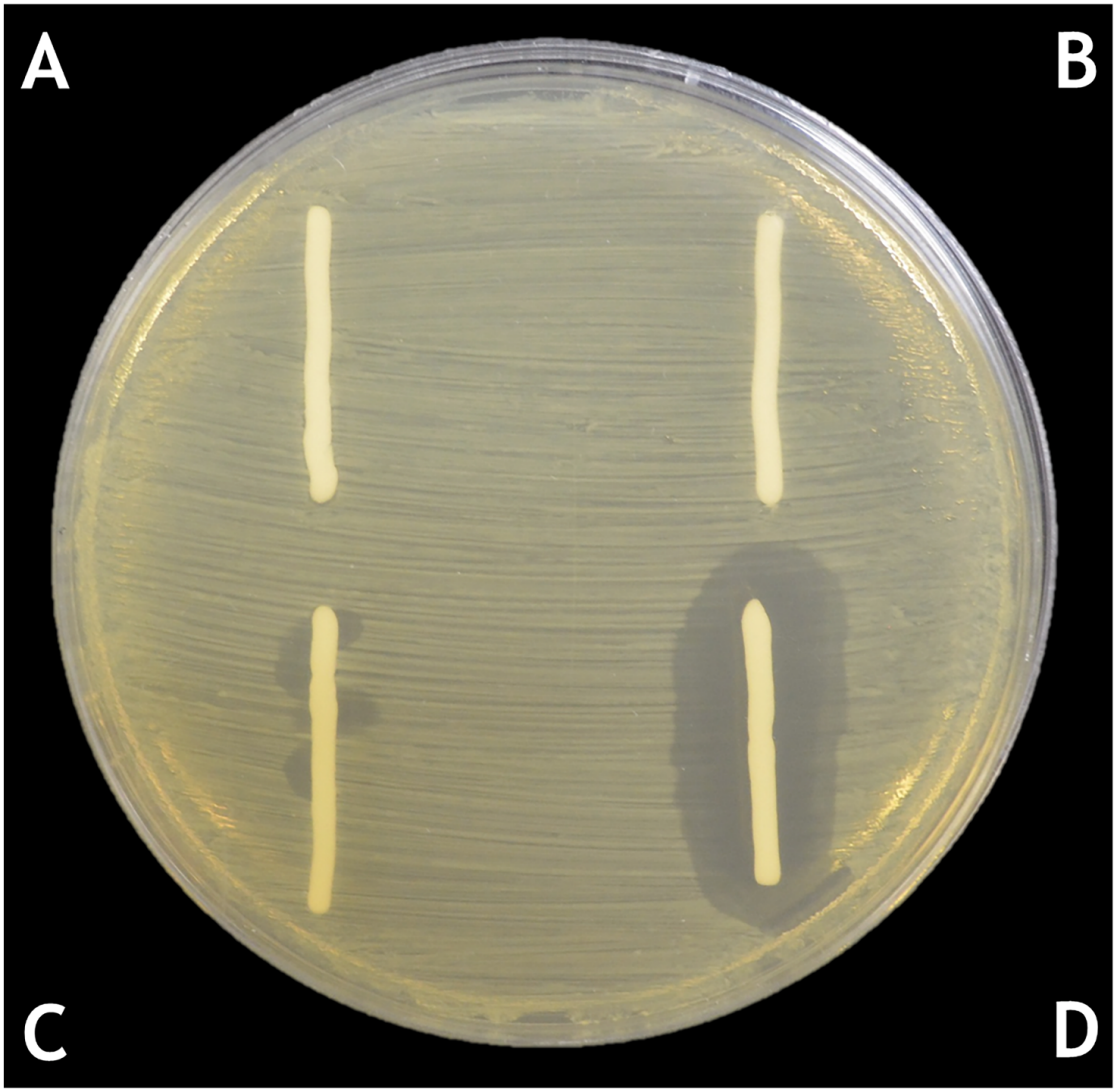
Lytic capacity of the *P*. *acnes* phage cream formulation. All PAC1 to PAC10 phage displayed similar behaviour when formulated in cetomacrogol cream aqueous and placed onto *P*. *acnes* lawns. Fig 6 shows results of experiments of cream formulated with PAC1 phage placed onto one of the *P*. *acnes* cutaneous strains isolated in this study. Legend: A. Cetomacrogol cream aqueous; B. Cetomacrogol cream aqueous with PBS; C. Phage cream, PAC1 at a concentration of 5.0x10^3^ PFU per gram of cream D. Phage cream, PAC1 at a concentration of 2.5x10^8^ PFU per gram of cream.

The ten PAC phage displayed similar behaviour when formulated in cetomacrogol cream aqueous and placed onto *P*. *acnes* lawns. In these formulations, they killed the surrounding bacteria forming an area of clearing. Fig 6 shows results of experiments when cream formulated with PAC1 phage was placed onto one of the *P*. *acnes* strains isolated in this study. The minimum phage concentration at which plaques were observed was at 5.0x10^3^ PFU per gram of cream. Maximal clearing was observed when 2.5x10^8^ PFU per gram of cream was used (Fig 6). The use of a cocktail of phage in the cream did not result in more extensive clearing than using a single phage at this concentration.

**Fig 7:**
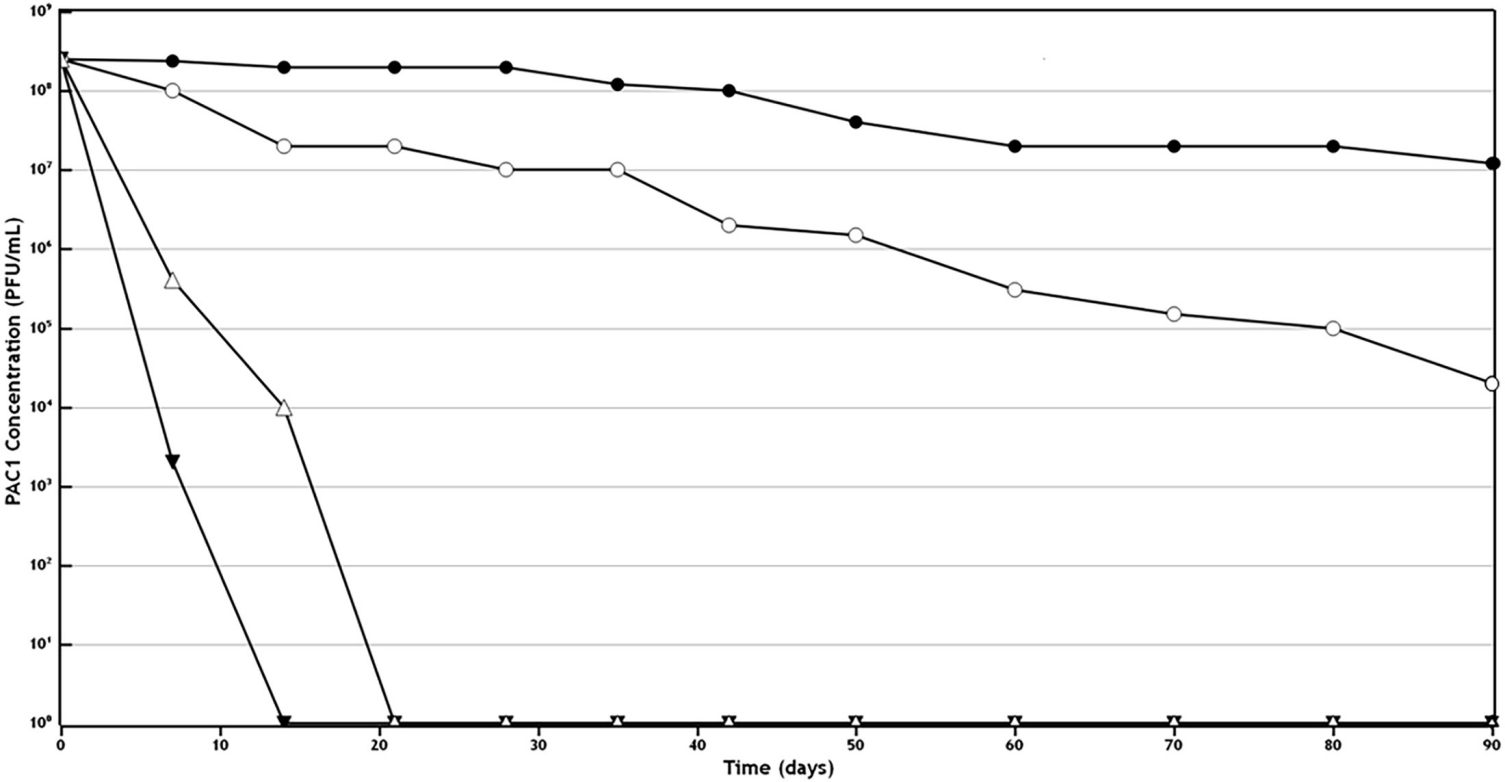
Quantitative assessment of the phage lytic activity and stability following storage of the creams at various temperatures and light exposures: 0.05 g samples of the phage cream were taken at weekly intervals, mixed into PBS to a volume of 1 mL, vortexed and centrifuged. An aliquot of a dilution series of the supernatant was then placed onto a lawn of *P*. *acnes* so as to ascertain the numbers of viable phage. Storage at 4°C in a light protected bottle resulted in optimal stability of the cream and efficacy of the phage activity. ● indicates storage at 4°C in a light protected bottle. **○** indicates storage at 20–25°C in a light protected bottle. ▼ indicates storage at 45°C in a light protected bottle. Δ indicates storage in constant light at 20–25°C.

Quantitative assessment of the phage lytic activity and stability following storage of the creams at various temperatures and light exposures showed that storage at 45°C resulted in loss of lytic capacity by 14 days. If the cream was stored at 25°C, then exposure to full light resulted in loss of lytic capacity by 21 days, while if protected from light, the phage lytic activity was maintained even after 90 days. Of all the conditions tested, storage at 4°C in a light protected bottle resulted in optimal stability of the cream and efficacy of the phage activity (Fig 7; S2 Table).

## Discussion

This study has shown that phage when applied in the form of a cream were able to lyse *P*. *acnes* isolates and this ability was maintained after prolonged storage of the supplemented cream. This is not the first report of isolation of phage active against this organism, but is the first time their potential for possible treatment of acne has been explored.

Characterisation of the PAC phage genomes revealed that they contained no known bacterial virulence factors, which if present may have impacted upon their potential application as therapeutic agents. None carried an integrase gene, and all could circularise. The *P*. *acnes* (ATCC 6919) strain developed immunity to subsequent lysis by these phage following initial exposure to them and resistant cells were shown to harbour phage DNA that could potentially be in the form of an episome. These genomic attributes lend weight to the suggestion that these phage have a pseudolysogenic life cycle as suggested for other *P*. *acnes* phage [41], particularly those infecting members of the IA_1_ clade [43], to which all *P*. *acnes* strains isolated in this study belong. How this feature may impact upon their use as therapeutic agents is discussed later.

### Phylogenetic comparisons between PAC phage and other *P*. *acnes* phage

The ten phage reported here are most closely linked evolutionarily to phage P101A (Fig 4). Comparing genomic data from the PAC phage to those of the other 62 *P*. *acnes* phage shows that their clustering pattern indicates 53 distinct subgroups, or strains (Fig 4) after branches with bootstrap values <80 were collapsed [43].

### Clinical application of phage and lytic capacity of the *P*. *acnes* phage cream formulations

*P*. *acnes* clade IA_1_ members are isolated frequently from acne lesions [9–13], and these bacteria infected with phage are thought to display a pseudolysogenic life cycle as defined by Liu et al. (2015). Consequently, as these infected bacteria then acquire superinfection immunity [43], the value of phage therapy using phage lytic for them may appear limited. As shown here and previously [43], plaques produced after phage lysis of the clade IA_1_ *P*. *acnes* (ATCC 6919) showed regrowth from their centre, consistent with the cells developing resistance [43]. Yet all of type IA_1_ *P*. *acnes* strains we isolated, were lysed completely by the 10 PAC phage. When they were applied to *P*. *acnes* cells as creams they all remained active for long periods if stored under appropriate temperatures and in the dark. Thus, the results presented here suggest that the phage applied as cream formulations to skin may provide a simple and efficient method for treating *P*. *acnes* skin lesions.

The benefit of a phage cream for *P*. *acnes* infection is that it reduces the impact of harmful side effects seen with the currently available topical and oral treatments for acne vulgaris. Cetomacrogol cream aqueous is used widely as a skin moisturiser to treat sensitive and dry skin [50], and as a base for incorporation of pharmaceutically active components. Its formulation with phage is likely to contribute to a moisturising and “soothing” of the skin, while allowing close contact of the phage to areas where *P*. *acnes* is actively growing.

Thus, data presented here suggest that the formulation, prescription and therapeutic application of this treatment is realistic in terms of its pharmaceutical stability, in contrast to a cream that requires frequent formulation. Recent studies suggest that it is possible to develop microgel delivery systems which are able to penetrate sebum and thus capable of delivering medicaments in close proximity to inflammatory processes within the hair follicle [4]. Such a carrier could conceivably convey a range of therapeutic agents, should they prove to be stable. It is important to note, however, that just as the success or failure of some pharmaceutical drugs may depend upon pharmacogenetics, or how the patient’s genetic profile affects drug disposition, the success of *P*. *acnes* phage cream applications in humans may depend upon the individual patient’s skin microbiome [37,43], and indeed, on whether some of the cutaneous bacteria may develop some level of immunity following exposure to phage therapy. In this instance, it may be necessary, following the initial reduction of bacterial numbers, to implement established topical or oral therapies to eliminate the remaining bacterial cells. Finally, although the use of a cocktail of phage in the experiments described here did not result in more extensive clearing than using a single phage, such an approach could be useful in combatting potential resistance issues, should they arise.

## Conclusion

We report here the isolation, genome sequencing and characterisation of ten bacteriophage able to lyse *P*. *acnes*, but no other *Propionibacterium* species. While displaying almost 98% homology to each other at the nucleotide level, their genomes show slight differences in their structural and functional genes compared to those described for other *P*. *acnes* phage. When the phage were formulated into a semi solid cream base they were capable of killing *P*. *acnes* bacteria, even after the formulation was stored for up to 90 days. These findings provide a model system for testing the efficacy of diverse phage against other pathogenic skin bacterial strains, and for subsequent application to humans.

## Supporting Information

S1 Table *P*. *acnes* bacteriophage aligned to generate the phylogenetic tree (Fig 4). (DOCX)

S2 Table Quantitation of phage lytic capacity following storage of phage cream under various conditions. Each experiment was performed in triplicate and values represent the average phage numbers, expressed as Plaque Forming Units (PFU) per mL. These data were used to generate Fig 7.
(DOCX)
