# Supplementary material for: The Formulation of Bacteriophage in a Semi Solid Preparation for Control of Propionibacterium acnes Growth
Source: PLoS One. 2016 Mar 10;11(3):e0151184. doi: 10.1371/journal.pone.0151184 (PMC4786141; doi:10.1371/journal.pone.0151184)
Supplement: S1 Table — (DOCX) [file pone.0151184.s001.docx]

**S1 Table**. *P. acnes* bacteriophage aligned to generate the phylogenetic tree (Fig 4).

| Phage | Accession number | Reference | |  | Phage | Accession number | | | Reference | |
| --- | --- | --- | --- | --- | --- | --- | --- | --- | --- | --- |
| ATCC29399B_C | JX262225.1 | Marinelli et al. 2012 | |  | PHL067M01 | KJ578765.1 | | | Lui et al. 2015 | |
| ATCC29399B_T | JX262224.1 | Marinelli et al. 2012 | |  | PHL067M09 | KJ578766.1 | | | Lui et al. 2015 | |
| P1.1 | JX262223.1 | Marinelli et al. 2012 | |  | PHL067M10 | JX570709.1 | | | Lui et al. 2015 | |
| P100.1 | JX262222.1 | Marinelli et al. 2012 | |  | PHL070N00 | KJ578767.1 | | | Lui et al. 2015 | |
| P100A | JX262221.1 | Marinelli et al. 2012 | |  | PHL071N05 | JX570710.1 | | | Lui et al. 2015 | |
| P100D | JX262220.1 | Marinelli et al. 2012 | |  | PHL073M02 | JX570703.1 | | | Lui et al. 2015 | |
| P101A | JX262217.1 | Marinelli et al. 2012 | |  | PHL082M00 | KJ578768.1 | | | Lui et al. 2015 | |
| P104A | JX262218.1 | Marinelli et al. 2012 | |  | PHL082M02 | KJ578769.1 | | | Lui et al. 2015 | |
| P105 | JX262219.1 | Marinelli et al. 2012 | |  | PHL082M03 | KJ578770.1 | | | Lui et al. 2015 | |
| P14.4 | JX262216.1 | Marinelli et al. 2012 | |  | PHL082M04 | KJ578771.1 | | | Lui et al. 2015 | |
| P9.1 | JX262215.1 | Marinelli et al. 2012 | |  | PHL085M01 | JX570707.1 | | | Lui et al. 2015 | |
| PA6 | DQ431235.1 | Farrar et al. 2007 |  | | PHL085N00 | | KJ578772.1 | Lui et al. 2015 | |  |
| PAC1 | KR902978 | This study | |  | PHL092M00 | KJ578773.1 | | | Lui et al. 2015 | |
| PAC2 | KR902979 | This study | |  | PHL095N00 | KJ578774.1 | | | Lui et al. 2015 | |
| PAC3 | KR902980 | This study | |  | PHL111M01 | JX570702.1 | | | Lui et al. 2015 | |
| PAC4 | KR902981 | This study | |  | PHL112N00 | JX570714.1 | | | Lui et al. 2015 | |
| PAC5 | KR902982 | This study | |  | PHL113M01 | JX570713.1 | | | Lui et al. 2015 | |
| PAC6 | KR902983 | This study | |  | PHL114L00 | JX570712.1 | | | Lui et al. 2015 | |
| PAC7 | KR902984 | This study | |  | PHL114N00 | KJ578775.1 | | | Lui et al. 2015 | |
| PAC8 | KR902985 | This study | |  | PHL115M02 | JX570708.1 | | | Lui et al. 2015 | |
| PAC9 | KR902986 | This study | |  | PHL116M00 | KJ578776.1 | | | Lui et al. 2015 | |
| PAC10 | KR902987 | This study | |  | PHL116M10 | KJ578777.1 | | | Lui et al. 2015 | |
| PAD20 | FJ706171.1 | Lood & Collin 2011 | |  | PHL117M00 | KJ578778.1 | | | Lui et al. 2015 | |
| PAS50 | FJ706172.1 | Lood & Collin 2011 | |  | PHL117M01 | KJ578779.1 | | | Lui et al. 2015 | |
| PHL009M11 | KJ578758.1 | Lui et al. 2015 | |  | PHL132N00 | KJ578780.1 | | | Lui et al. 2015 | |
| PHL010M04 | JX570704.1 | Lui et al. 2015 | |  | PHL141N00 | KJ578781.1 | | | Lui et al. 2015 | |
| PHL025M00 | KJ578759.1 | Lui et al. 2015 | |  | PHL150M00 | KJ578782.1 | | | Lui et al. 2015 | |
| PHL030N00 | KJ578760.1 | Lui et al. 2015 | |  | PHL151M00 | KJ578783.1 | | | Lui et al. 2015 | |
| PHL037M02 | JX570706.1 | Lui et al. 2015 | |  | PHL151N00 | KJ578784.1 | | | Lui et al. 2015 | |
| PHL041M10 | KJ578761.1 | Lui et al. 2015 | |  | PHL152M00 | KJ578785.1 | | | Lui et al. 2015 | |
| PHL055N00 | KJ578762.1 | Lui et al. 2015 | |  | PHL163M00 | KJ578786.1 | | | Lui et al. 2015 | |
| PHL060L00 | JX570705.1 | Lui et al. 2015 | |  | PHL171M01 | KJ578787.1 | | | Lui et al. 2015 | |
| PHL064M01 | KJ578763.1 | Lui et al. 2015 | |  | PHL179M00 | KJ578788.1 | | | Lui et al. 2015 | |
| PHL064M02 | KJ578764.1 | Lui et al. 2015 | |  | PHL194M00 | KJ578789.1 | | | Lui et al. 2015 | |
| PHL066M04 | JX570711.1 | Lui et al. 2015 | |  | PHL199M00 | KJ578790.1 | | | Lui et al. 2015 | |
