# Supplementary material for: The Formulation of Bacteriophage in a Semi Solid Preparation for Control of Propionibacterium acnes Growth
Source: PLoS One. 2016 Mar 10;11(3):e0151184. doi: 10.1371/journal.pone.0151184 (PMC4786141; doi:10.1371/journal.pone.0151184)
Supplement: S2 Table — Each experiment was performed in triplicate and values represent the average phage numbers, expressed as Plaque Forming Units (PFU) per mL. These data were used to generate Fig 7. (DOCX) [file pone.0151184.s002.docx]

**Table S2**. Quantitation of phage lytic capacity following storage of phage cream under various conditions. Each experiment was performed in triplicate and values represent the average phage numbers, expressed as Plaque Forming Units (PFU) per mL. These data were used to generate Fig 7.

| Time | 4°C (light protected bottle) | 20- 25°C (light protected bottle) | 45°C (light protected bottle) | Constant light (20-25°C) |
| --- | --- | --- | --- | --- |
| Day 1 | 2.5 x 10^8^ PFU/mL | 2.5 x 10^8^ PFU/mL | 2.5 x 10^8^ PFU/mL | 2.5 x 10^8^ PFU/mL |
| Day 7 | 2.4 x 10^8^ PFU/mL | 1.0 x 10^8^ PFU/mL | 2.0 x 10^3^ PFU/mL | 4.0 x 10^5^ PFU/mL |
| Day 14 | 2.0 x 10^8^ PFU/mL | 2.0 x 10^7^ PFU/mL | No plaques | 1.0 x 10^4^ PFU/mL |
| Day 21 | 2.0 x 10^8^ PFU/mL | 2.0 x 10^7^ PFU/mL | No plaques | No plaques |
| Day 28 | 2.0 x 10^8^ PFU/mL | 1.0 x 10^7^ PFU/mL | No plaques | No plaques |
| Day 35 | 1.2 x 10^8^ PFU/mL | 1.0 x 10^7^ PFU/mL | No plaques | No plaques |
| Day 42 | 1.0 x 10^8^ PFU/mL | 2.0 x 10^6^ PFU/mL | No plaques | No plaques |
| Day 50 | 4.0 x 10^7^ PFU/mL | 1.5 x 10^6^ PFU/mL | No plaques | No plaques |
| Day 60 | 2.0 x 10^7^ PFU/mL | 3.0 x 10^5^ PFU/mL | No plaques | No plaques |
| Day 70 | 2.0 x 10^7^ PFU/mL | 1.5 x 10^5^ PFU/mL | No plaques | No plaques |
| Day 80 | 2.0 x 10^7^ PFU/mL | 1.0 x 10^5^ PFU/mL | No plaques | No plaques |
| Day 90 | 1.2 x 10^7^ PFU/mL | 2.0 x 10^4^ PFU/mL | No plaques | No plaques |
